# Supplementary material for: Multielemental, Nutritional, and Proteomic Characterization of Different Lupinus spp. Genotypes: A Source of Nutrients for Dietary Use
Source: Molecules. 2022 Dec 10;27(24):8771. doi: 10.3390/molecules27248771 (PMC9787123; doi:10.3390/molecules27248771)
Supplement: Supplementary file 1 [file molecules-27-08771-s001.zip › molecules-2069866-Supplementary Figures.pdf]

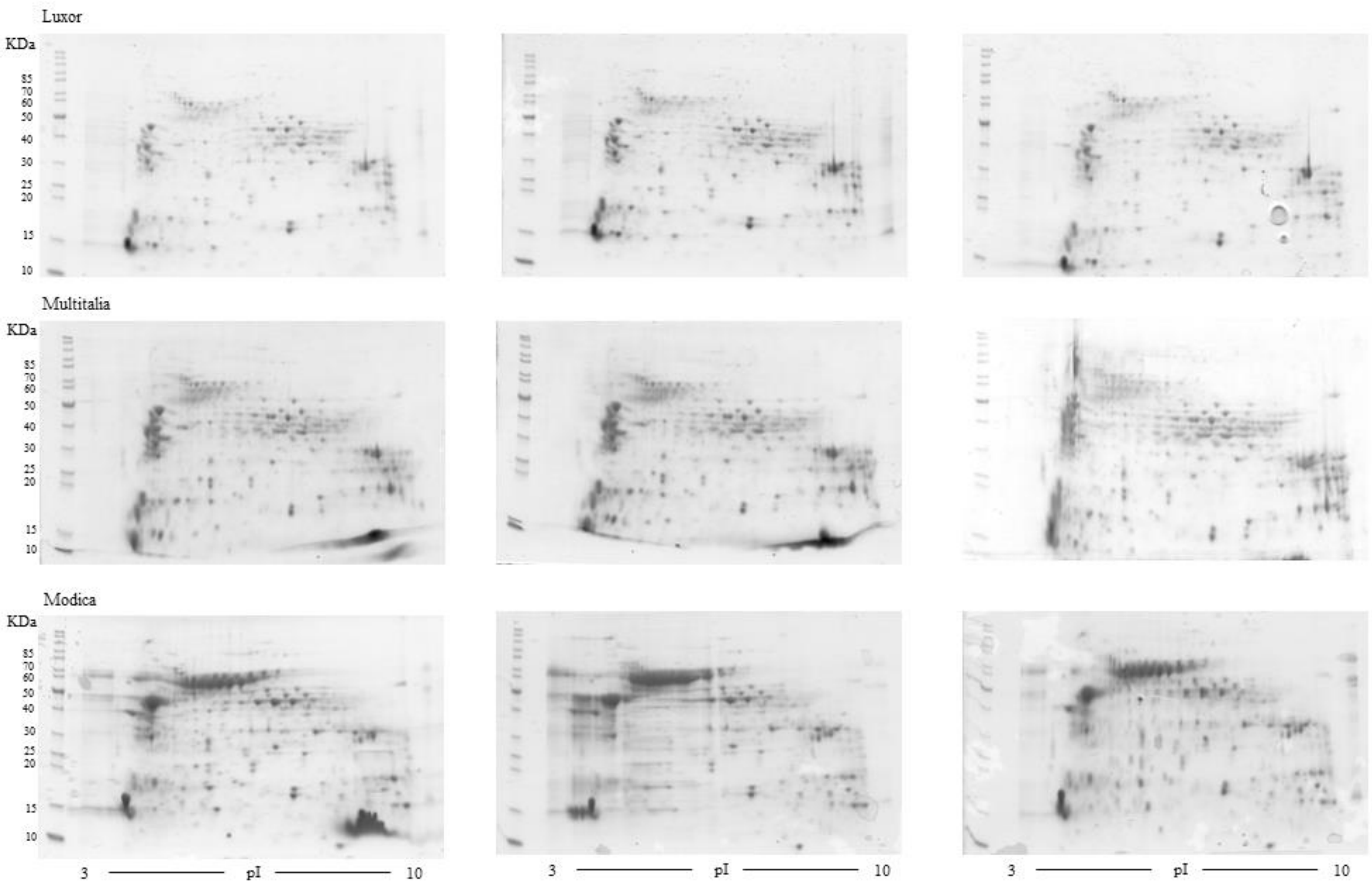

Figure S1. 2D gel replicates of *Lupinus albus* cultivars (Luxor, Multitalia and Modica Ecotype)

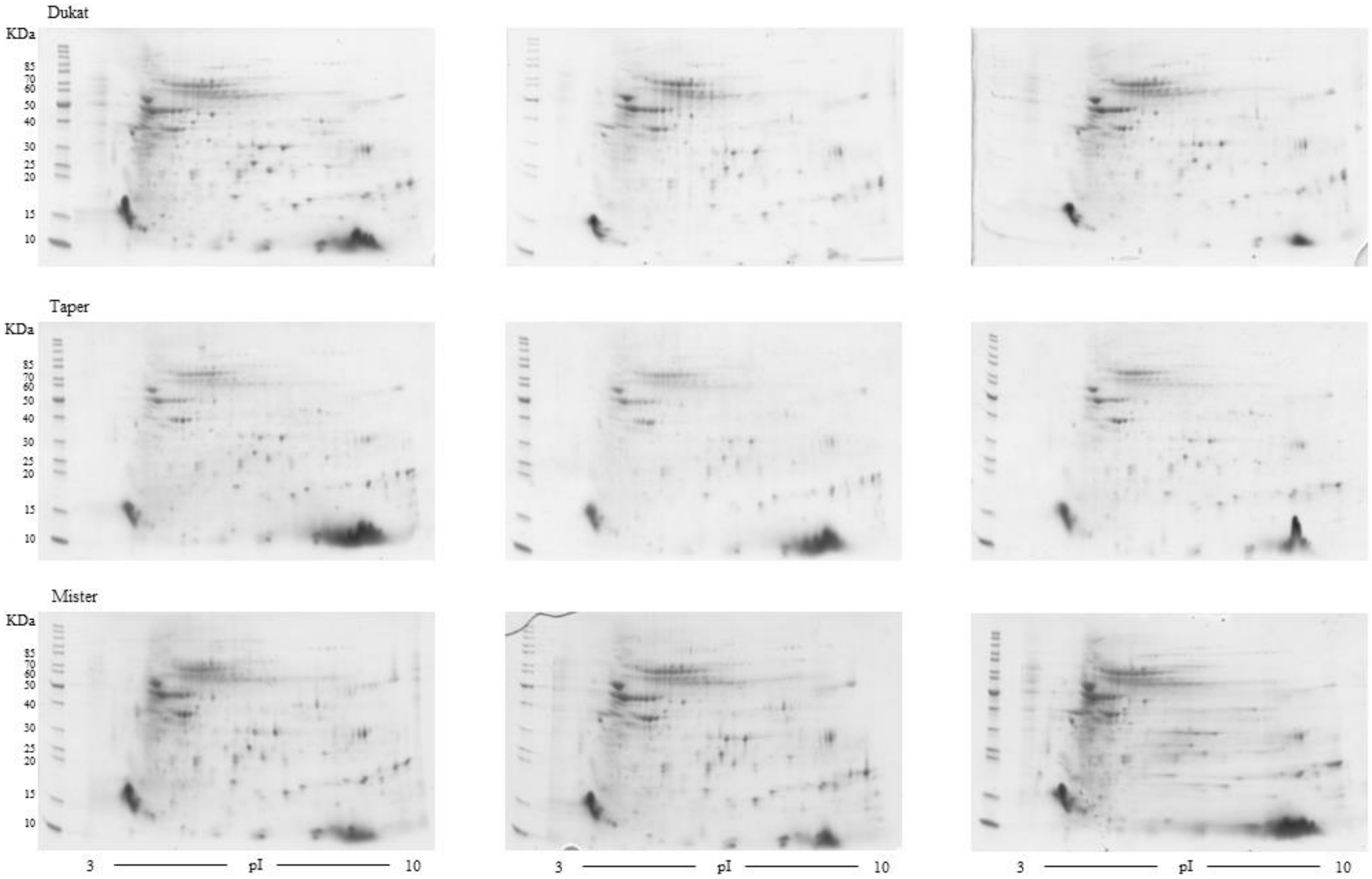

Figure S2. 2D gel replicates of *Lupinus luteus* cultivars (Dukat, Taper and Mister)

Figure S3. 2D gel replicates of *Lupinus angustifolius* cultivar (Sonet)

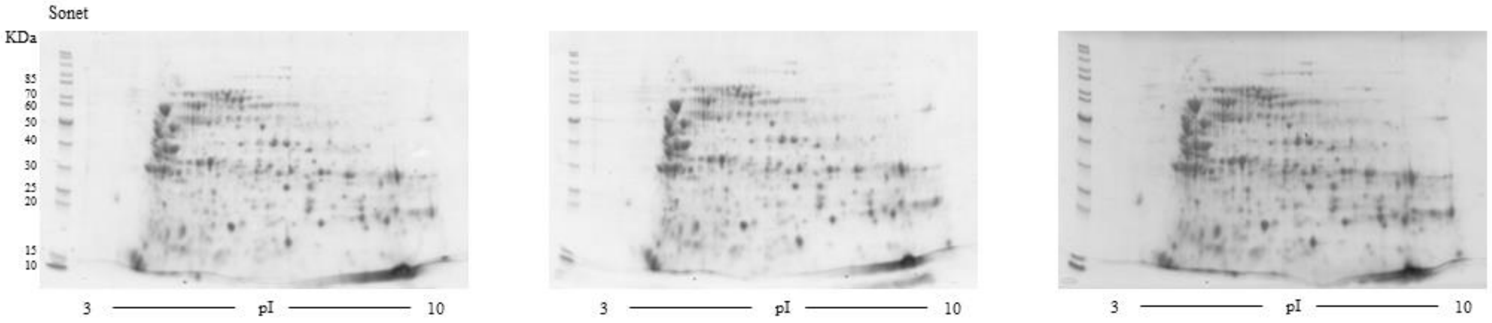

```
sp|F5B8W0|CONB2_LUPAN      MANMRVKFPTLVLLLGIVFLMAVSIGIAYGEKNAIKNHERPQEREQEERDPRQQPRPRHQ 60
sp|Q6EBC1|CONB2_LUPAL      MGKMRVRFPTLVLVLGIVFLMAVSIGIAYGEKDVLKSHERPEEREQ-------------- 46
sp|Q53HY0|CONB1_LUPAL      MGKMRVRFPTLVLVLGIVFLMAVSIGIAYGEKDVLKSHERPEEREQ-------------- 46
                           *.:***:******:******************:.:*.****:****

sp|F5B8W0|CONB2_LUPAN      EEQEREHGREEERNREPSRGRSESEESREEEREQRREPSRGREQEQQPQHGRREEEEEWQ 120
sp|Q6EBC1|CONB2_LUPAL      --------------------------------------------------------EEWQ 50
sp|Q53HY0|CONB1_LUPAL      --------------------------------------------------------EEWQ 50
                                                                                   ****

sp|F5B8W0|CONB2_LUPAN      PRRQRPQSRREEREQ--EQGSSSSSGRQSGYERR------EQREEREQQQEQDSRSESRR 172
sp|Q6EBC1|CONB2_LUPAL      PRRQRPQSRREEREQEQEQGSPSYPRRQSGYERRQYHERSEQREEREQEQQQGSPSYSRR 110
sp|Q53HY0|CONB1_LUPAL      PRRQRPQSRREEREQEQEQGSPSYPRRQSGYERRQYHERSEQREEREQEQQQGSPSYSRR 110
                           ***************  **** *   ********      ********:*:*.* * ***

sp|F5B8W0|CONB2_LUPAN      QRNPYYFSYERFQTLYKNRNGQIRVLERFDQRTNRLENLQNYRIVEFQSKPNTLILPKHS 232
sp|Q6EBC1|CONB2_LUPAL      QRNPYHFSSQRFQTLYKNRNGKIRVLERFDQRTNRLENLQNYRIVEFQSKPNTLILPKHS 170
sp|Q53HY0|CONB1_LUPAL      QRNPYHFNSQRFQTLYKNRNGKIRVLERFDQRTNRLENLQNYRIVEFQSKPNTLILPKHS 170
                           *****:*. :***********:**************************************

sp|F5B8W0|CONB2_LUPAN      DADYILVVLNGRATITIVNPDKRQAYNLEHGDALRLPAGTTSYILNPDDNQNLRVVKLAI 292
sp|Q6EBC1|CONB2_LUPAL      DADYVLVVLNGRATITIVNPDRRQAYNLEYGDALRIPAGSTSYILNPDDNQKLRVVKLAI 230
sp|Q53HY0|CONB1_LUPAL      DADYVLVVLNGRATITIVNPDRRQAYNLEYGDALRIPAGSTSYILNPDDNQKLRVVKLAI 230
                           ****:****************:*******:*****:***:***********:********

sp|F5B8W0|CONB2_LUPAN      PINNPGNFYDFYPSSTKDQQSYFNGFSRNTLEATFNTRYEEIQRIILGNEDGQEDEEQSR 352
sp|Q6EBC1|CONB2_LUPAL      PINNPGYFYDFYPSSTKDQQSYFSGFSRNTLEATFNTRYEEIQRIILGNEDEQEYEEQRR 290
sp|Q53HY0|CONB1_LUPAL      PINNPGYFYDFYPSSTKDQQSYFSGFSRNTLEATFNTRYEEIQRILLGNEDEQEYEEQRR 290
                           ****** ****************.*********************:***** ** *** *

sp|F5B8W0|CONB2_LUPAN      GQEQSHQDQGVIVRVSKEQIQELRKHAQSSSGKGKPSESGPFNLRSDEPIYSNKFGNFYE 412
sp|Q6EBC1|CONB2_LUPAL      GQEQSDQDEGVIVIVSKKQIQKLTKHAQSSSGKDKPSDSGPFNLRSNEPIYSNKYGNFYE 350
sp|Q53HY0|CONB1_LUPAL      GQEQSHQDEGVIVRVSREQIQELTKYAQSSSGKDKPSQSGPFNLRSNEPIYSNKYGNFYE 350
                           *****.**:**** **::***:* *:*******.***:********:*******:*****

sp|F5B8W0|CONB2_LUPAN      ITPDRNPQAQDLDISLTFIEINEGGLLLPHYNSKAIFVVVVDEGEGNYELVGIRDQERQQ 472
sp|Q6EBC1|CONB2_LUPAL      ITPDRNPQVQDLNISLTYIKINEGALLLPHYNSKAIYVVVVDEGEGNYELVGIRDQQRQQ 410
sp|Q53HY0|CONB1_LUPAL      ITPDRNPQVQDLDISLTFTEINEGALLLPHYNSKAIFIVVVGEGNGKYELVGIRDQQRQQ 410
                           ********.***:****: :****.***********::***.**:*:*********:***

sp|F5B8W0|CONB2_LUPAN      DEQE--QEEVRRYNAKLSEGDIFVIPAGHPISINASSNLRLLGFGINADENQRNFLAGSE 530
sp|Q6EBC1|CONB2_LUPAL      DEQEEKEEEVIRYSARLSEGDIFVIPAGYPISINASSNLRLLGFGINADENQRNFLAGSK 470
sp|Q53HY0|CONB1_LUPAL      DEQEEEPEEVRRYSARLSEGDIFVIPAGYPISVNASSNLRLLGFGINAYENQRNFLAGSE 470
                           ****   *** **.*:************:***:*************** **********:


sp|F5B8W0|CONB2_LUPAN      DNVIRQLDKEVKQLTFPGSVEDVERLIKNQQQSYFANAQPQQQQ--QREKEGRRGRRGLS 588
sp|Q6EBC1|CONB2_LUPAL      DNVIRQLDRAVNELTFPGSAEDIERLIKNQQQSYFANGQPQQQQQQQSEKEGRRGRRGSS 530
sp|Q53HY0|CONB1_LUPAL      DNVIRQLDREVKELTFPGSAEDIERLIKNQQQSYFANALPQQQ--QQSEKEGRRGRRGPI 528
                           ********: *::******.**:**************. ****   * **********

sp|F5B8W0|CONB2_LUPAN      FPFRSLFTKLLSTIM      603
sp|Q6EBC1|CONB2_LUPAL      LPF------------      533
sp|Q53HY0|CONB1_LUPAL      SSI------------      531
                             :
```

Figure S4. Sequence alignment of β-globulin/vicilin F5B8W0, Q6EBC1, and Q53HY0.

```
tr|Q53I54|Q53I54_LUPAL      MSNKLLALSLFLPFLLLFFCGCFASTFRQQPQENECQFQRLNALEPDNTVQSEAGTIETW        60
tr|Q53I55|Q53I55_LUPAL      ------------------------------------------------------------        0

tr|Q53I54|Q53I54_LUPAL      NPKNDELRCAGVALSRCTIQRNGLRRPFYTNAPQEIYIQQGRGIFGMIFPGCGETYEEPQ        120
tr|Q53I55|Q53I55_LUPAL      ------------------------------------------------------------        0

tr|Q53I54|Q53I54_LUPAL      ESEKGQGPRPQDRHQKVEHFKEGDIIAVPTGIPFWMYNDGQTPVVAITLIDTTNLDNQLD        180
tr|Q53I55|Q53I55_LUPAL      ------------------------------------------------------------        0

tr|Q53I54|Q53I54_LUPAL      QIPRRFYLSGNQEQEFLQYQEKEGGQGQQQEGGNVLSGFDDEFLEEALSVNKEIVRNIKG        240
tr|Q53I55|Q53I55_LUPAL      ------------------------------------------------------------        0

tr|Q53I54|Q53I54_LUPAL      KNDDREGGIVEVKGGLKVIIPPTMRPRHGREEEEEEEEEDERRGDRRRRHPHHHHHEEEE        300
tr|Q53I55|Q53I55_LUPAL      ----------PVNRQLRSV----------------FPGRPTRPGDRRRRHPHHHHHEEEE        34
                                       *:  *: :                   .  * *****************

tr|Q53I54|Q53I54_LUPAL      EEEEEWSHQVRRVRRPHRHHHHRKDRNGLEETLCTMKLRHNIGESTSPDAYNPQAGRFKT        360
tr|Q53I55|Q53I55_LUPAL      EEEEEWSHQVRRVRRPHRHHHHRKDRNGLEETLCTMKLRHNIGESTSPDAYNPQAGRFKT        94
                            ************************************************************

tr|Q53I54|Q53I54_LUPAL      LTSIDFPILGWLGLAAEHGSIYKNALFVPYYNVNANSILYVLNGSAWFQVVDCSGNAVFN        420
tr|Q53I55|Q53I55_LUPAL      LTSIDFPILGWLGLAAEHGSN-KNALFVPYYNVNANSILYVLNGSAWFQVVDCSGNAVFN        153
                            ********************  **************************************

tr|Q53I54|Q53I54_LUPAL      GELNEGQVLTIPQNYAAAIKSLSDNFRYVAFKTNDIPQIATLAGANSEISALPLEVVAHA        480
tr|Q53I55|Q53I55_LUPAL      GELNEGQVLTIPQNYAAAIKSLSDNFRYVAFKTNDIPQIATLAGANSEISALPLEVVAHA        213
                            ************************************************************

tr|Q53I54|Q53I54_LUPAL      FNLNRDQARQLKNNNPYKFLVPPPQSQLRAVA    512
tr|Q53I55|Q53I55_LUPAL      FNLNRAQARQLKNTNPYKFLVPPPQSQLRAVA    245
                            ***** *******.*******************
```

Figure S5. Sequence alignment of legumin-like proteins Q53I54 and Q53I55.

```
sp|F5B8V6|CONA1_LUPAN      MANK-LLALS--LFLL-FSGCFASTFRQQPQQNECQFQRLNALEPDNSVKSEAGTIETWN 56
sp|F5B8V7|CONA2_LUPAN      MAKPCLFSFSLCLLLL-SSLCLAE---RPERYKECQLDRLNALEPDNRVESEGGVTETWN 56
sp|F5B8V8|CONA3_LUPAN      MANPFLLSLSLCLVLLYTSACLGE---GLDRFNECQLDRLNALEPDNRIESEGGVTETWN 57
                           **:  *:::*  *.**  * *:..      : :***::********* ::**.*. ****

sp|F5B8V6|CONA1_LUPAN      PNNDQLRCAGVALSRCTIQRNGLRRPFYTNAPQEIYIQQGRGIFGLIFPGCRETYEEPQE 116
sp|F5B8V7|CONA2_LUPAN      SNRPELRCAGVAFEKHTIQPQGLHLPSYTNYPQLIFIVEGEGALGISVPGCTETYEEAQQ 116
sp|F5B8V8|CONA3_LUPAN      SNKPELRCAGVAFEKHTIEPKGLHLPSYTNYPQIIMIVQGEGALGISVPGCTETFEEAQQ 117
                            *. :*******:.: **: :**: * *** ** * * :*.* :*: .*** **:** *:

sp|F5B8V6|CONA1_LUPAN      QEQGQGP------------RPQDRHQKVEHFREGDIIAVPTGVPFWMYNNEQTPVIAITL 164
sp|F5B8V7|CONA2_LUPAN      SQSSQDPRRRSSRSQSQEQEQQDSHQKIQYFREGDIIAIPPGIPYWTYNYGEQRLVAINL 176
sp|F5B8V8|CONA3_LUPAN      SQSRQERRRG---QRSQSQEQEDSHQKIRHFREGDILVIPPGTPYWTYNYGDEQLVAINL 174
                           .:. *              . :* ***:.:******:.:* * *:* **  :  ::**.*

sp|F5B8V6|CONA1_LUPAN      IDTTNLDNQLDQIPRRFYLSGNQEQEFLQYQQKEGGQGQQQ--------------EGGNE 210
sp|F5B8V7|CONA2_LUPAN      LDTTSLLNQLDPSPRRFYIAGNPEEEHPETQEQQGQQREQQQGAGGRRRGKHQQEQEEEG 236
sp|F5B8V8|CONA3_LUPAN      LDTTSLSNQLDPNPRRFYLAGNPEEEYPETQQQR-QQRQQHQRPSGRRHGQHQKEEEQEG 233
                           :***.* ****  *****::** *:*. : *::.  * :*:              :  :

sp|F5B8V6|CONA1_LUPAN      GGNVLSGFNDEFLEEAFSVDREIVRNIKGKNDDREGSIVEVKEGLKVISPPTLRPRQGRE 270
sp|F5B8V7|CONA2_LUPAN      KNNVLSGFDPQFLTQAFNVDEEIINRLQNP-DERLKQIVRVKRGLSIISPKSQEEEEE-- 293
sp|F5B8V8|CONA3_LUPAN      KNNILSGFDPQFLSQALNIDEDTVHKLQNP-NERIKQIIRVEEGLGVISPKWQEQEEEEE 292
                            .*:****: :** :*:.:*.: :..::.  ::*  .*:.*:.** :***   . .:

sp|F5B8V6|CONA1_LUPAN      EE-----------------E-EEEEEEEERRGDRRR------HR------PHHH-EEE- 297
sp|F5B8V7|CONA2_LUPAN      EEEEPRQRGQPERREERREEEKEEEEEEDEPRSRERYERQSRRRPGRQQGRQGEEQEEES 353
sp|F5B8V8|CONA3_LUPAN      EKEEPRQRRRRERREEREEEEKEE---EDEPRESRR-------HR------GGHE-EEEV 335
                           *:                  * **   *:* *  .*       :         .. ***

sp|F5B8V6|CONA1_LUPAN      -------EEEEEWSHQVRRV---------------------------------------- 310
sp|F5B8V7|CONA2_LUPAN      ESEQEGRGQQREWERTTRHRRAQGEEGEEEEEETSTRVRRQQGRGRGQEQGQEQGQEQEQ 413
sp|F5B8V8|CONA3_LUPAN      EEERGRGRGGSEWKRTTRRRHTRGDEGQEEEETTTTTEE--------------------- 374
                                      **.: .*:

sp|F5B8V6|CONA1_LUPAN      ---------------------------------RRPHHHREDRNGLEETLCTLKLRHNIG 337
sp|F5B8V7|CONA2_LUPAN      EEEQQEGRRGRHGGRGRRSGQQREEEEEEQQQQGRRKRQESRNGLEETICTARLLENIA 473
sp|F5B8V8|CONA3_LUPAN      -------RRRRRGGRGSRQEE-----E-------EEQSPPRSRNGLEETICTAILRENIA 415
                                                               :   ..*******:**  * .**.

sp|F5B8V6|CONA1_LUPAN      QSTSPDAYNPQAGRLKTLTSLDFPILRWLGLAAEHGSIYKNAMFVPYYNVNANSILYVLN 397
sp|F5B8V7|CONA2_LUPAN      KPSRADLYNPNAGRISSVNSLTLPILRWFQLSADYVNLYRNGIYAPHWNINANSVIFVTR 533
sp|F5B8V8|CONA3_LUPAN      DPTRADLYNPTAGRISTANSLTLPILGWFQLSAEYVNLYRNGIYAPHWNINANSVIYVIR 475
                           . :  * *** ***:.: .** :*** *: *:*:: .:*:*.::.*::*:****:::* .

sp|F5B8V6|CONA1_LUPAN      GSAWFQVVDCSGNAVFNGELNEGQVLTIPQNYAVAIKSLDDNFSYVAFKTNDIPQIAALA 457
sp|F5B8V7|CONA2_LUPAN      GRGRVQVVNCQGNSVFNDDLRRGQLLVVPQNFVVAHQAGDEGFEFIAFKTNDLAATSP-- 591
sp|F5B8V8|CONA3_LUPAN      GRGRVQVVNSQGNSVFNDDLRRGQLLVVPQNFVVAHQAGDEGFEFIAFKTNDQATTSP-- 533
                           * . .***:..**:***.:*..**:*.:***:.** :: *:.*.::******    :

sp|F5B8V6|CONA1_LUPAN      GLTSSIRALPLDVVAHAFNLDRDQARQLKNNNPYKFLVPPPQ-SQLRAV---A        506
sp|F5B8V7|CONA2_LUPAN      -VKQVFRGIPAEVLANAFGLRLNQVSQLKYSGNQGPLVSPQSESEDHTLPKVA        643
sp|F5B8V8|CONA3_LUPAN      -LKQVFRGIPAEVLANAFRLSLNQVSELKYNGNHNPLVTPQSQSQDHNLVKVA        585
                            :.. :*.:* :*:*:** *  :*. :** ..    ** * . *: : :   *
```

Figure S6. Sequence alignment of α-globulin proteins F5B8V6, F5B8V7 and F5B8V8

```
tr|B0YJF8|B0YJF8_LUPAN      MAKMRVRLPMLILLLGVVFLLAASIGIAYGEKDFTKNP--PKEREEEEHEPRQQPRPRQQ      58
sp|F5B8V9|CONB1_LUPAN       MAKMRVRLPMLILLLGVVFLLAASIGIAYGEKDFTKNP--PKEREEEEHEPRQQPRPRQQ      58
tr|B8Q5G0|B8Q5G0_LUPAN      MAKMRVRLPMLILLLGVVFLLAASIGIAYGEKDFTKNP--PKEREEEEHEPRQQPRPRQQ      58
sp|F5B8W3|CONB5_LUPAN       MAKMRVRFPMLVLLLGVVFLLAVSIGIAYGEKDVIKNPERPEERQEEERDPRQPPRSRQQ      60
tr|B0YJF7|B0YJF7_LUPAN      ------------------------------------------------------------      0
sp|F5B8W0|CONB2_LUPAN       MANMRVKFPTLVLLLGIVFLMAVSIGIAYGEKNAIKNHERPQEREQEERDPRQQPRPRHQ      60
sp|F5B8W1|CONB3_LUPAN       MAKMRVRFPTLVLLLGIVFLMAVSIGIAYGEKNVLKNHERPQEREQEERDPRQQPRPHHQ      60
sp|F5B8W2|CONB4_LUPAN       MIKMRVRFPTLVLLLGIVFLMAVSIGIAYGEKNVIKNHERPQEREQEERDPRQQPRPHHQ      60

tr|B0YJF8|B0YJF8_LUPAN      EEQEREHRREEKHDGEPSRGRSQSEESQEEEHERRREHHREREQEQQPRPQRRQEEE---      115
sp|F5B8V9|CONB1_LUPAN       EEQEREHRREEKHDGEPSRGRSQSEESQEEEHERRREHHREREQEQQPRPQRRQEEE---      115
tr|B8Q5G0|B8Q5G0_LUPAN      EEQEREHRREEKHDGEPSRGRSQSEESQEEEHERRREHHREREQEQQPRPQRRQEEE---      115
sp|F5B8W3|CONB5_LUPAN       EEQEREHRREKERDREPSRGRSESKQSQEEERERRKEHDREREQEQQPQYGRRHEEEEKG      120
tr|B0YJF7|B0YJF7_LUPAN      ------------------------EERQEEERERRREPCREREQEQQPQHGRREEEE---      33
sp|F5B8W0|CONB2_LUPAN       EEQEREHGREEERNREPSRGRSESEESREEEREQRREPSRGREQEQQPQHGRREE-----      115
sp|F5B8W1|CONB3_LUPAN       EEQEREHRRES-------------EESQEEEREQRREPRREREQEQQPQHGRREE-----      102
sp|F5B8W2|CONB4_LUPAN       EEQEREHRREEERDREPSRGRSESEESREEEREQRREPRREREQEQQPQHGRREE-----      115
                                                    :: :***:*:*:*  * *******:  **.*

tr|B0YJF8|B0YJF8_LUPAN      EEEEEWQPRRQRPQSRREEREEREQEQGSSSGSQRGSGDERRQH----------------      159
sp|F5B8V9|CONB1_LUPAN       EEEEEWQPRRQRPQSRREEREEREQEQGSSSGSQRGSGDERRQH----------------      159
tr|B8Q5G0|B8Q5G0_LUPAN      EEEEEWQPRRQRPQSRREEREEREQEQGSSSGSQRGGGDERRQH----------------      159
sp|F5B8W3|CONB5_LUPAN       EEEEEGQARRQRPQRRREE---REQEQGSSSESRRQSGDERRHRHEKREQREEREQEQGS      177
tr|B0YJF7|B0YJF7_LUPAN      -EEEEWQPRRLRPQSRKEE---REQEQGSSSSSRKQSGYERRQY----------------      73
sp|F5B8W0|CONB2_LUPAN       --EEEWQPRRQRPQSRREE---REQEQGSSSSSGRQSGYERREQ----------------      154
sp|F5B8W1|CONB3_LUPAN       --EEEWQPRRQRPQSRREE---REQEQGSSSSSRRQSGYERREQ----------------      141
sp|F5B8W2|CONB4_LUPAN       --EEEWQPRRQRPQSRREE---REQEQGSSSSSRRQSGYERRE-----------------      153
                              *** * ** *** *:**   ********* * : .* ***.

tr|B0YJF8|B0YJF8_LUPAN      -------------RERRVHREEREQEQDSRSDSRRQRNPYHFSSNRFQTYYRNRNGQIRV      206
sp|F5B8V9|CONB1_LUPAN       -------------RERRVHREEREQEQDSRSDSRRQRNPYHFSSNRFQTYYRNRNGQIRV      206
tr|B8Q5G0|B8Q5G0_LUPAN      -------------RERRVHREEREQEQDSRSDSRRQRNPYHFSSNRFQTYYRNRNGQIRV      206
sp|F5B8W3|CONB5_LUPAN       SSGRQSDYGRRQRHEGREQREEREQEQGSSSESHRLRNPYYFSSERFQTRYKNKNGQIRV      237
tr|B0YJF7|B0YJF7_LUPAN      -------------HERREQRDEKEKEQDSRSDSRRQRNPYHFSSERFQTRYRNRNGQIRV      120
sp|F5B8W0|CONB2_LUPAN       -------------REEREQ----QQEQDSRSESRRQRNPYYFSYERFQTLYKNRNGQIRV      197
sp|F5B8W1|CONB3_LUPAN       -------------REEREQ----EQEQGSRSDSRRQRNPYYFSSERFQTLYRNRNGQIRV      184
sp|F5B8W2|CONB4_LUPAN       ---------------EREQ----EQEQGSRSDSRRQRNPYYFSSERFQTLYRNRNGQIRV      194
                                            * :    ::**.* *:*:* ****:** :**** *:*:******

tr|B0YJF8|B0YJF8_LUPAN      LERFNQRTNRLENLQNYRIIEFQSKPNTLILPKHSDADFILVVLNGRATITIVNPDKRQV      266
sp|F5B8V9|CONB1_LUPAN       LERFNQRTNRLENLQNYRIIEFQSKPNTLILPKHSDADFILVVLNGRATITIVNPDKRQV      266
tr|B8Q5G0|B8Q5G0_LUPAN      LERFNQRTNRLENLQNYRIIEFQSKPNTLILPKHSDADFILVVLNGRATITIVNPDKRQV      266
sp|F5B8W3|CONB5_LUPAN       LERFDQRTNRLENLQNYRIVEFQSRPNTLILPKHSDADYILVVLNGRATITIVNPDKRQA      297
tr|B0YJF7|B0YJF7_LUPAN      LERFDQRTNRLENLQNYRIVEFQSNPNTLILPKHSDADYILVVLNGRATITIVNPDKRQA      180
sp|F5B8W0|CONB2_LUPAN       LERFDQRTNRLENLQNYRIVEFQSKPNTLILPKHSDADYILVVLNGRATITIVNPDKRQA      257
sp|F5B8W1|CONB3_LUPAN       LERFDQRTNRLENLQNYRIVEFQSKPNTLILPKHSDADYILVVLNGSATITIVNPDKRQS      244
sp|F5B8W2|CONB4_LUPAN       LERFDQRTDRLENLQNYRIVEFQSKPNTLILPKHSDADYILVVLNGSATITIVNPDKRQS      254
                            ****:***:**********:****.*************:******* ************

tr|B0YJF8|B0YJF8_LUPAN      YNLEQGDALRLPAGTTSYILNPDDNQNLRVAKLAIPINNPGKLYDFYPSTTKDQQSYFSG      326
sp|F5B8V9|CONB1_LUPAN       YNLEQGDALRLPAGTTSYILNPDDNQNLRVAKLAIPINNPGKLYDFYPSTTKDQQSYFSG      326
tr|B8Q5G0|B8Q5G0_LUPAN      YNLEQGDALRLPAGTTSYILNPDDNQNLRVAKLAIPINNPGKLYDFYPSTTKDQQSYFSG      326
sp|F5B8W3|CONB5_LUPAN       YNLEYGDALRLPAGTTSYILNPDDNQDLRVVKLAIPINNPGKFYDFYPSRTKDQQSYFSG      357
tr|B0YJF7|B0YJF7_LUPAN      YNLEYGDALRLPAGTTSYILNPDDNQNLRVVKLAIPINNPSNFYDFYPSSTKDQQSYFSG      240
sp|F5B8W0|CONB2_LUPAN       YNLEHGDALRLPAGTTSYILNPDDNQNLRVVKLAIPINNPGNFYDFYPSSTKDQQSYFNG      317
sp|F5B8W1|CONB3_LUPAN       YNLENGDALRLPAGTTSYILNPDDNQNLRVVKLAIPINNPGNFYDFYPSSSKDQQSYFSG      304
sp|F5B8W2|CONB4_LUPAN       YNLENGDALRLPAGTTSYILNPDDNQNLRVVKLAIPINNPGNFYDFYPSSSKDQQSYFSG      314
                            **** *********************:***.*********.::****** :*******.*

tr|B0YJF8|B0YJF8_LUPAN      FSKNTLEATFNTRYEEIERVLLGDDELQENEKQRRGQEQSHQDEGVIVRVSKKQIQELRK      386
sp|F5B8V9|CONB1_LUPAN       FSKNTLEATFNTRYEEIERVLLGDDELQENEKQRRGQEQSHQDEGVIVRVSKKQIQELRK      386
tr|B8Q5G0|B8Q5G0_LUPAN      FSKNTLEATFNTRYEEIERVLLGDDELQENEKQRRGQEQSHQDEGVIVRVSKKQIQELRK      386
sp|F5B8W3|CONB5_LUPAN       FSKNTLEATFNTHYEEIQRILLGYEDEQEDEEQRREQEQSHQDEGVIVRVSKEQIQELRK      417
tr|B0YJF7|B0YJF7_LUPAN      FSKNTLEATFNTRYEEIQRILLGNEDEQEDEEQRRGQEQSYQDEGVIVRVSKEQIQELRK      300
sp|F5B8W0|CONB2_LUPAN       FSRNTLEATFNTRYEEIQRIILGNEDGQEDEEQSRGQEQSHQDQGVIVRVSKEQIQELRK      377
sp|F5B8W1|CONB3_LUPAN       FSKNTLEATFNTRYEEIQSILLGNEDEQEDDEQWHGQEQSHQDEGVIVRVSKEQVQELRK      364
sp|F5B8W2|CONB4_LUPAN       FSRNTLEATFNTRYEEIQRILLGNEDEQEDDEQRHGQEQSHQDEGVIVRVSKEQVQELRK      374
                            **:*********:****: ::** :: **:::* : ****:**:********:*:*****

tr|B0YJF8|B0YJF8_LUPAN      HAQSSSGEGKPSESGPFNLRSNKPIYSNKFGNFYEITPDINPQFQDLNISLTFTEINELW      446
sp|F5B8V9|CONB1_LUPAN       HAQSSSGEGKPSESGPFNLRSNKPIYSNKFGNFYEITPDINPQFQDLNISLTFTEINEGA      446
tr|B8Q5G0|B8Q5G0_LUPAN      HAQSSSGEGKPSESGPFNLRSNKPIYSNKFGNFYEITPDINPQFQDLNISLTFTEINEGA      446
sp|F5B8W3|CONB5_LUPAN       HAQSSSRKGKPSESGPFNLRSNEPIYSNKFGNFYEITPDRNPQVQDLDISLIFTEISEGA      477
tr|B0YJF7|B0YJF7_LUPAN      HAQSSSRKGKPSESGPFNLRSNESIYSNKFGNFYEITPERNPQVQDLDISLTFTEINEGA      360
sp|F5B8W0|CONB2_LUPAN       HAQSSSGKGKPSESGPFNLRSDEPIYSNKFGNFYEITPDRNPQAQDLDISLTFIEINEGG      437
sp|F5B8W1|CONB3_LUPAN       YAQSSSRKGKPYESGPFNLRSNKPIYSNKFGNFYEITPDRNPQAQDLDISLTFIEINEGA      424
sp|F5B8W2|CONB4_LUPAN       YAQSSSRKGKPSKSGPFNLRSNKPIYSNKFGNFYEITPNRNPQAQDLDISLTFIEINEGA      434
                            :***** :*** :********:: **************: *** ***:*** * **.*

tr|B0YJF8|B0YJF8_LUPAN      LTKEKE----IMN-----------------------------------------------      455
sp|F5B8V9|CONB1_LUPAN       LLLPHYNSKAIFIVVVDEGEGNYELVGIRDQQRQQDEQEEEYEQGEEEVRRYSDKLSKGD      506
tr|B8Q5G0|B8Q5G0_LUPAN      LLLPHYNSKAIFIVVVDEGEGNYELVGIRDQQRQQDEQEEEYEQGEEEVRRYSDKLSKGD      506
sp|F5B8W3|CONB5_LUPAN       LLLPHYNSKAIFVIVVDEGEGNYELVGIRNQQRQQDEQE------VEEVRSYNARLSEGD      531
tr|B0YJF7|B0YJF7_LUPAN      LLLPHYNSKAIFIVVVDEGEGNYELVGIRDQQRQQDEQEEE----EEEVRRYSARLSEGD      416
sp|F5B8W0|CONB2_LUPAN       LLLPHYNSKAIFVVVVDEGEGNYELVGIRDQERQQDEQE------QEEVRRYNAKLSEGD      491
sp|F5B8W1|CONB3_LUPAN       LLLPHYNSKAIFVVVVDEGEGNYELVGIRDQQRQQDEQ---------EVRRYSARLSEGD      475
sp|F5B8W2|CONB4_LUPAN       LLLPHYNSKAIFVVLVDEGEGNYELVGIRDQQRQQDEQ---------EVRRYSARLSEGD      485
                            *   :     *:

tr|B0YJF8|B0YJF8_LUPAN      ------------------------------------------------------------      455
sp|F5B8V9|CONB1_LUPAN       VFIIPAGHPLSINASSNLRLLGFGINANENQRNFLAGSEDNVIKQLDREVKELTFPGSIE      566
tr|B8Q5G0|B8Q5G0_LUPAN      VFIIPAGHPLSINASSNLRLLGFGINANENQRNFLAGSEDNVIKQLDREVKELTFPGSIE      566
sp|F5B8W3|CONB5_LUPAN       ILVIPAGHPLSINASSNLRLLGFGINADENQRNFLAGSEDNVIRQLDREVKELIFPGSAE      591
tr|B0YJF7|B0YJF7_LUPAN      IFVIPAGYPISVNASSNLRLLGFGINANENQRNFLAGSEDNVISQLDREVKELTFPGSAQ      476
sp|F5B8W0|CONB2_LUPAN       IFVIPAGHPISINASSNLRLLGFGINADENQRNFLAGSEDNVIRQLDKEVKQLTFPGSVE      551
sp|F5B8W1|CONB3_LUPAN       IFVIPAGHPISINASSNLRLLGFGINADENQRNFLAGSEDNVIRQLDREVKGLIFPGSAE      535
sp|F5B8W2|CONB4_LUPAN       IFVIPAGHPISINASSNLRLLGFGINADENQRNFLAGSEDNVIRQLDTEVKGLTFPGSTE      545

tr|B0YJF8|B0YJF8_LUPAN      -----------------------------------------------------      455
sp|F5B8V9|CONB1_LUPAN       DVERLIKNQQQSYFANAQPQQQ-QQREKEGRRGRRGPISSILNALY-------      611
tr|B8Q5G0|B8Q5G0_LUPAN      DVERLIKNQQQSYFANAQPQQQ-QQREKEGRRGRRGPISSILNALY-------      611
sp|F5B8W3|CONB5_LUPAN       DVERLIRNQQQSYFANAQPQQQQQREKEGRRGRRGPISSILSALY-------      637
tr|B0YJF7|B0YJF7_LUPAN      DVERLIKNQQQSYFANAQPQQK-QQREKEGRRGRRSLISSILSTL-------Y      521
sp|F5B8W0|CONB2_LUPAN       DVERLIKNQQQSYFANAQPQQQ-QQREKEGRRGRRGLSFPFRSLFTKLLSTIM      603
sp|F5B8W1|CONB3_LUPAN       DVERLIKNQQQSYFANAQPQQQ-QQREREGRHGRRGHISSILSTLY-------      580
sp|F5B8W2|CONB4_LUPAN       DVERLIKNQQQSYFANAQPQQQ-QQREREGRRGRRGHISSILSTLY-------      590
```

Figure S7. Sequence alignment of β-globulin proteins B0YJF7, B0YJF8, F5B8W0, F5B8W1, F5B8W2, F5B8W3, F5B8V9 and B8Q5G0.

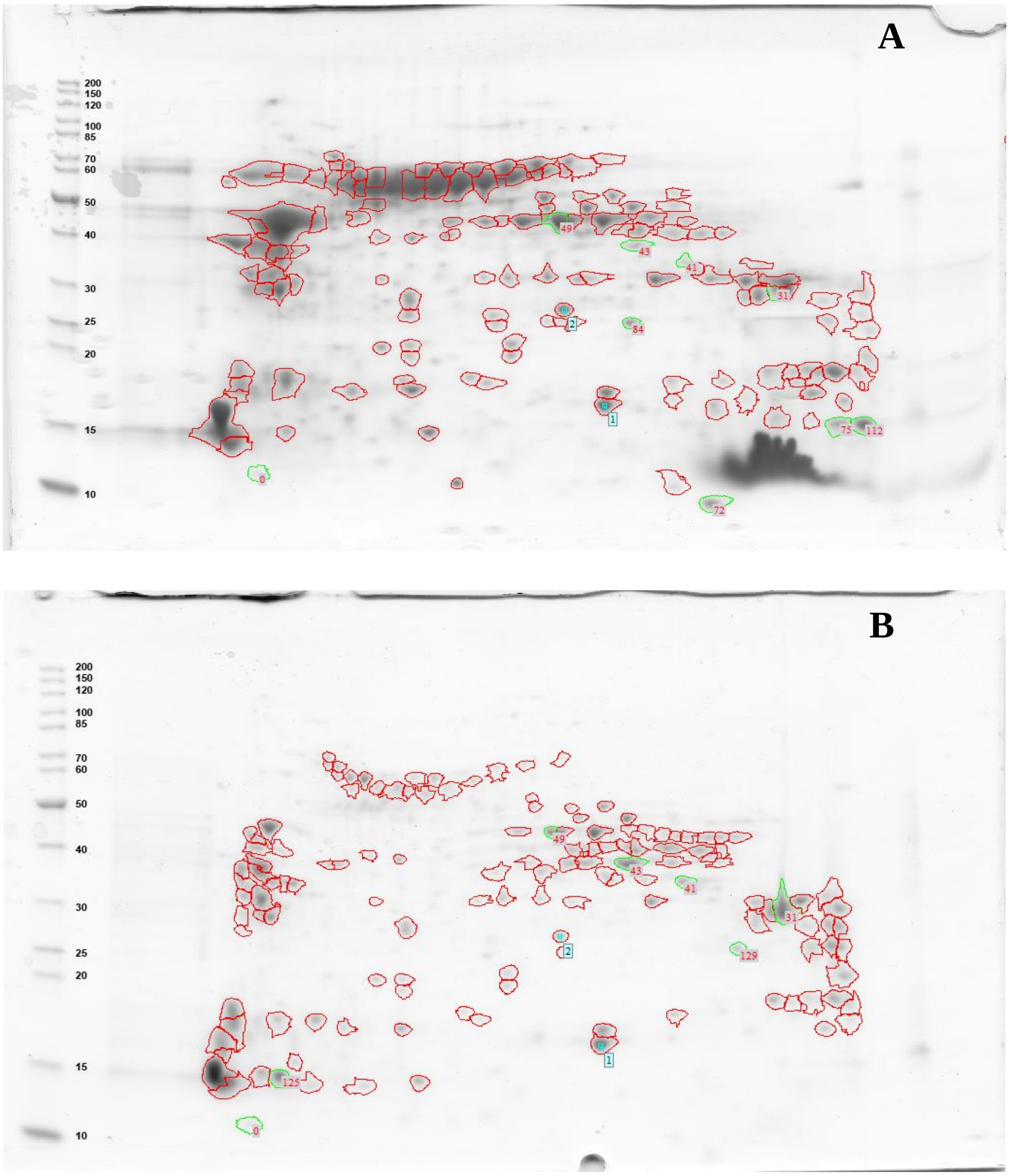

Figure S8. 2D gel map comparison of Modica Ecotype (A) and Luxor cultivar (B). Excised spots are highlighted in green. The corresponding identifications are reported in **Tables S2 – 4**, where spot ID are reported with an asterisk (*).

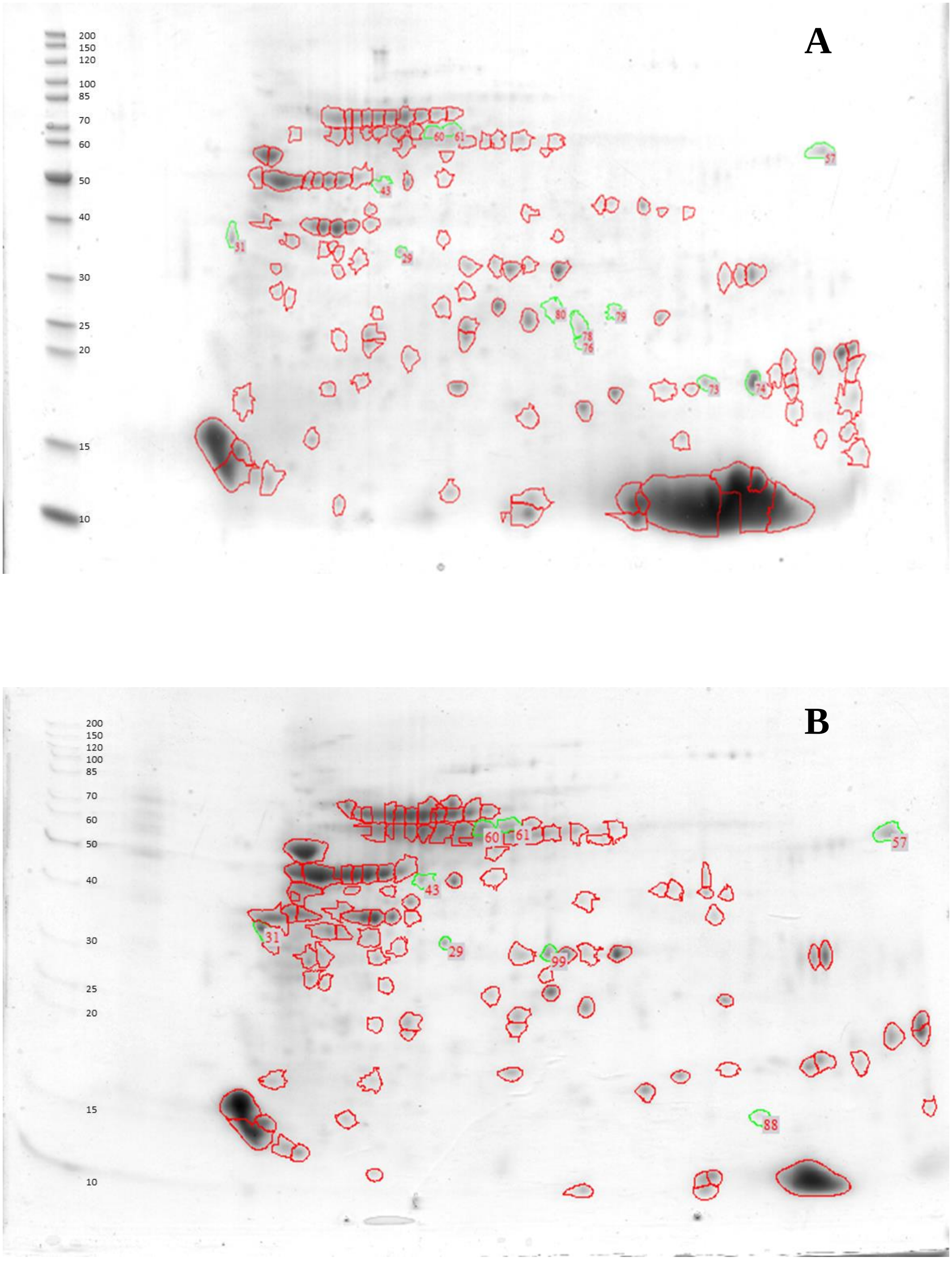

Figure S9. 2D gel map comparison of Taper (A) and Dukat cultivar (B). Excised spots are highlighted in green. The corresponding identifications are reported in **Tables S5 – 6**, where spot ID are reported with an asterisk (*).

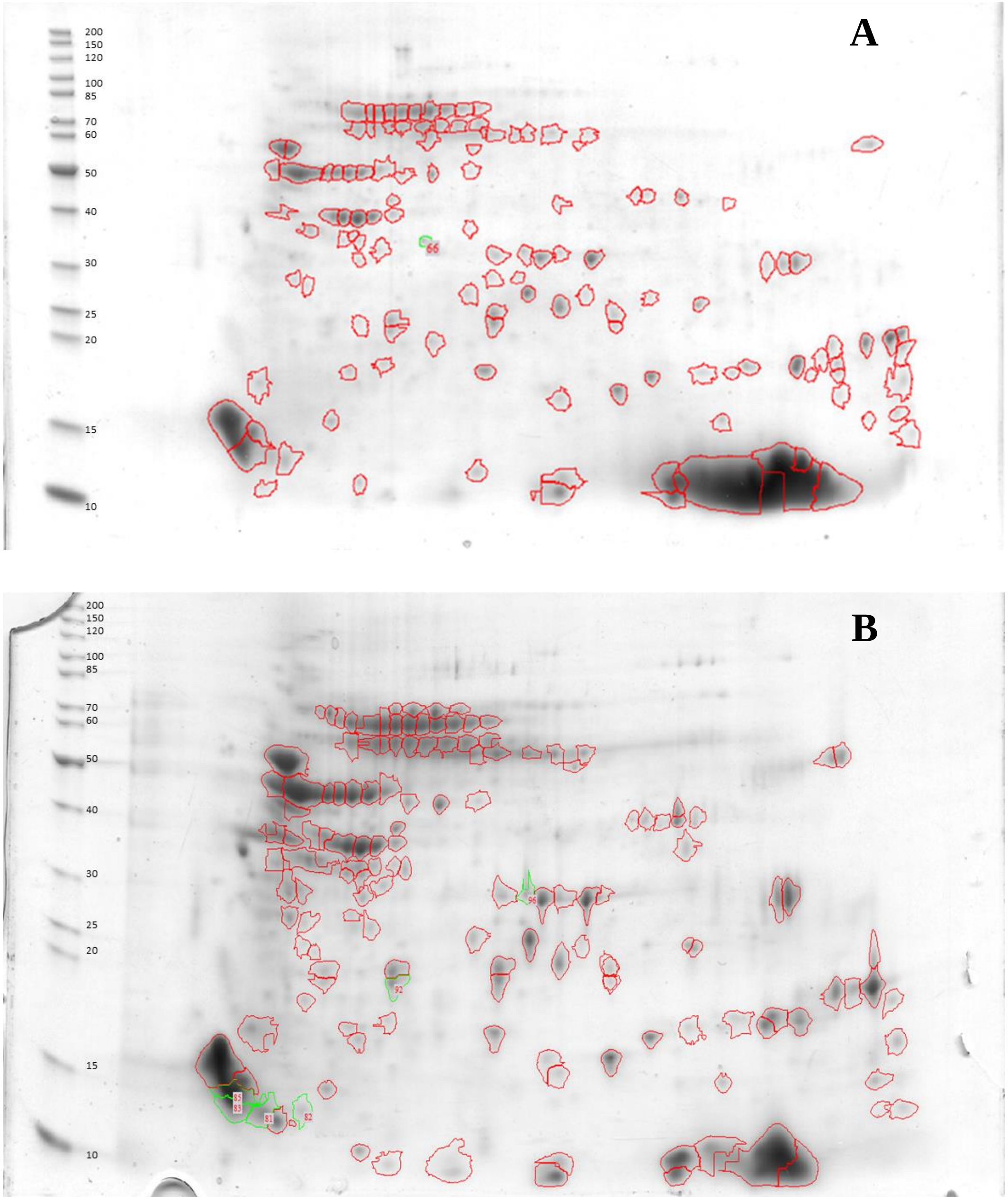

Figure S10. 2D gel map comparison of cv Taper (A) cv Mister (B). Excised spots are highlighted in green. The corresponding identifications are reported in **Tables S7 - 8**, where spot ID are reported with an asterisk (*).
