# Supplementary material for: Multielemental, Nutritional, and Proteomic Characterization of Different Lupinus spp. Genotypes: A Source of Nutrients for Dietary Use
Source: Molecules. 2022 Dec 10;27(24):8771. doi: 10.3390/molecules27248771 (PMC9787123; doi:10.3390/molecules27248771)
Supplement: Supplementary file 1 [file molecules-27-08771-s001.zip › molecules-2069866-Supplementary Tables.pdf]

Table S1. List of the identified protein spots in Luxor cultivar of *Lupinus albus*. Spots are referred to **Figure 3A**.

**Spot**	**Protein [Organism]**	**Accession Number**	**Coverage %**	**Exp Mr**	**Theor Mr/pI**	**MASCOT score**	**Sequence region (amino acid position)**
124	Conglutin β [*Lupinus albus*]	Q6EBC1	32	40	54.3/6.27	1119	Central (111-339)
125	Vicilin-like protein [*Lupinus albus*]	Q53HY0	27	35	62.0/6.08	1279	Central (143-315)
126	NI			83			
127	NI			83			
136	Vicilin-like protein (Fragment) [*Lupinus albus*]	Q53HY0	19	70	62.0/6.08	504	Central (154-464)
141/161	Vicilin-like protein (Fragment) [*Lupinus albus*]	Q53HY0	56	19	62.0/6.08	1966	
142/167/160	Conglutin β precursor [*Lupinus albus*]	Q6EBC1	26	69.5	62.1/6.43	1112	Central (97-384)
145	Conglutin β [*Lupinus albus*]	Q6EBC1	36	60	62.1/6.43	878	Central (64-495)
148	Conglutin β [*Lupinus albus*]	Q6EBC1	52	60	62.1/6.43	1606	Central (60-495)
149/163	Conglutin β [*Lupinus albus*]	Q6EBC1	36	65	62.1/6.43	878	Central (64-495)
154	Conglutin β precursor [*Lupinus albus*]	Q6EBC1	46	68.5	62.1/6.43	2408	Central and C-Terminal (143-289/427-495)
155	Vicilin-like protein (Fragment) [*Lupinus albus*]	Q53HY0	64	60	62.0/6.08	2201	Central and C-terminal (60-521)
158/147	Vicilin-like protein (Fragment) [*Lupinus albus*]	Q53HY0	61	52	62.0/6.08	1956	Central and C-terminal (60-521)
178/173	Vicilin-like protein (Fragment) [Lupinus albus]	Q53HY0	59	55	62.0/6.42	5474.75	
183/194	Vicilin-like protein (Fragment) [Lupinus albus]	Q6EBC1	35	50	62.0/6.42	1523.58	
188/189/192	Vicilin-like protein (Fragment) [*Lupinus albus*]	Q53HY0	58	50	62.0/6.08	1860	Central and C-terminal (90-521)
201	Conglutin β [*Lupinus albus*]	Q6EBC1	27	48	62.1/6.43	712	Central (154-463)
202	Conglutin α 2 [*Lupinus angustifolius*]	F5B8V7	45	48	74.6/5.12	3665	

202	Conglutin β [*Lupinus albus*]	Q6EBC1	18	49	62.1/6.43	386	Central (169-404)
204/211	Conglutin β [*Lupinus albus*]	Q6EBC1	48	50	62.1/6.43	1643	N-terminal and Central (45-408)
204/211	Conglutin γ [*Lupinus albus*]	Q9FSH9	21	50	49.9/8.39	373	
207/218	Vicilin-like protein (Fragment) [*Lupinus albus*]	Q53HY0	36	48	62.0/6.08	957	Central and C-terminal (154-521)
209	Vicilin-like protein (Fragment) [*Lupinus albus*]	Q53HY0	27	47	62.0/6.08	702	Central (154-463)
214	Conglutin β precursor [*Lupinus albus*]	Q6EBC1	24	44.5	62.1/6.43	1281	
223	Vicilin-like protein (Fragment) [*Lupinus albus*]	Q53HY0	45	48	62.0/6.08	1604	N-terminal and Central (45-408)
227	Conglutin α 2 [*Lupinus angustifolius*]	F5B8V7	6	42	74.6/5.12	268	C-terminal (556-611)
229	Putative quinone oxidoreductase [*Cicer arietinum*]	Q8L5Q7	22	20	21.7/6.51	245	Central (53-191)
231	Vicilin-like protein [*Lupinus albus*]	Q53HY0	24	40.5	62/6.08	1424	Central (143-315)
233	Legumin-like protein [*Lupinus albus*]	Q53I54	23	43	59.0/5.53	526	N-terminal and Central (29-253)
233	Vicilin-like protein (Fragment) [*Lupinus albus*]	Q53HY0	17	43	62.0/6.08	475	Central (154-463)
235/254	Legumin-like protein [*Lupinus albus*]	Q53I54	49	20	59.0/5.53	1150	N-terminal and Central (29-485)
238/265	Vicilin-like protein (Fragment) [*Lupinus albus*]	Q53HY0	31	43	62.0/6.08	771	Central (146-463)
240	Vicilin-like protein (Fragment) [*Lupinus albus*]	Q53HY0	19	41	62.0/6.08	525	Central (146-289)
245	Vicilin-like protein (Fragment) [*Lupinus albus*]	Q53HY0	43	40	61.9/6.08	1549	Central (60-404)
248	Vicilin-like protein (Fragment) [*Lupinus albus*]	Q53HY0	41	40	61.9/6.08	1226	Central (60-397)
256	Legumin-like protein [*Lupinus albus*]	Q53I54	47	38	59.0/5.53	2547	N-terminal and Central (29-485)
257/260	Vicilin-like protein (Fragment) [*Lupinus albus*]	Q53HY0	52	38	62.0/6.08	1985	N-terminal and Central (45-404)
257/260	Conglutin γ [*Lupinus albus*]	Q9FSH9	17	38	49.9/8.39	296	
261	Conglutin β [*Lupinus albus*]	Q6EBC1	50	38	62.1/6.43	2070	Central (63-408)

261	Conglutin γ [*Lupinus albus*]	Q9FSH9	21	38	49.9/8.39	396	
262	Vicilin-like protein (Fragment) [*Lupinus albus*]	Q53HY0	51	44	62.0/6.08	2016	Central (60-408)
262	Conglutin γ [*Lupinus albus*]	Q9FSH9	14	44	49.9/8.39	276	
263	Vicilin-like protein (Fragment) [*Lupinus albus*]	Q53HY0	36	45	61.9/6.08	1060	Central (64-335)
267/285/258	Vicilin-like protein (Fragment) [*Lupinus albus*]	Q53HY0	37	35	62.0/6.08	1048	Central and C-terminal (75-139)
268/269	Vicilin-like protein (Fragment) [*Lupinus albus*]	Q53HY0	40	38	62.0/6.08	1640	Central (90-404)
273	Vicilin-like protein [*Lupinus albus*]	Q53HY0	41	40	62.06.08	2293	Central (94-397)
274	Vicilin-like protein (Fragment) [*Lupinus albus*]	Q53HY0	40	37	62.0/6.08	1459	Central (90-408)
277	Conglutin α 1 [*Lupinus angustifolius*]	F5B8V6	47	31	58.1/5.26	2710	
278	Vicilin-like protein (Fragment) [*Lupinus albus*]	Q53HY0	40	35	61.9/6.08	1435	Central (90-404)
284	Conglutin β [*Lupinus albus*]	Q6EBC1	36	32	62.1/6.43	951	Central (154-495)
286	Vicilin-like protein (Fragment) [*Lupinus albus*]	Q53HY0	24	31	62.0/6.08	714	Central (228-495)
288/295/306	Vicilin-like protein (Fragment) [*Lupinus albus*]	Q53HY0	41	32	62.0/6.08	1199	Central (143-495)
294/293	Vicilin-like protein (Fragment) [*Lupinus albus*]	Q53HY0	45	33	62.0/6.08	1344	Central (143-463)
294/293	Conglutin γ [*Lupinus albus*]	Q9FSH9	27	33	49.9/8.39	643	
296	Vicilin-like protein (Fragment) [*Lupinus albus*]	Q53HY0	19	30	62.0/6.08	526	Central (143-345)
296	Conglutin δ Seed storage protein [*Lupinus albus*]	Q333K7	35	14	17.6/5.47	394	Central and C-terminal (75-131)
296	Conglutin γ [*Lupinus albus*]	Q9FSH9	14	14	49.9/8.39	335	Central and C-terminal (324-426)
297	Vicilin-like protein (Fragment) [*Lupinus albus*]	Q53HY0	39	30	62.0/6.08	1101	Central and C-terminal (206-521)
302	Conglutin β 2 [*Lupinus angustifolius*]	F5B8W0	23	30	70.7/5.64	376	
303	Vicilin-like protein (Fragment) [*Lupinus albus*]	Q53HY0	43	30	62.0/6.08	1280	Central and C-terminal (194-521)

308	Conglutin β [*Lupinus albus*]	Q6EBC1	38	30	62.1/6.43	1236	N-terminal and Central (51-451)
308	Conglutin γ [*Lupinus albus*]	Q9FSH9	11	30	49.9/8.39	288	Central (51-251)
309	Vicilin-like protein (Fragment) [*Lupinus albus*]	Q53HY0	41	30	61.9/6.08	1348	
309	Conglutin γ [*Lupinus albus*]	Q9FSH9	22	30	49.9/8.39	1348	Central and C-terminali (51-351/401-452)
310	Vicilin-like protein (Fragment) [*Lupinus albus*]	Q53HY0	36	30	61.9/6.08	1031	Central and C-terminal (101-301/451-521)
310	Conglutin γ [*Lupinus albus*]	Q9FSH9	33	30	49.9/8.39	641	Central and C-terminal (51-351/401-452)
311	Vicilin-like protein [*Lupinus albus*]	Q53HY0	26	30.5	62.0/6.08	732	
313	Conglutin β 2 [*Lupinus angustifolius*]	F5B8W0	31	27.5	70.7/5.64	1345	Central and C-terminal (175-576)
314	Conglutin β precursor [*Lupinus albus*]	Q6EBC1	7	27	62.1/6.43	305	Central (154-289)
315	Conglutin β [*Lupinus albus*]	Q6EBC1	31	30	62.1/6.43	1014	Central (101-451)
315	Conglutin γ [*Lupinus albus*]	Q9FSH9	9	30	49.9/8.39	201	Central (51-251)
317	Vicilin-like protein (Fragment) [*Lupinus albus*]	Q53HY0	36	29	61.9/6.08	1123	Central and C-terminal (101-301/451-531)
317	Conglutin γ [*Lupinus albus*]	Q9FSH9	16	29	49.9/8.39	341	Central and C-terminal (51-251/401-452)
320	Vicilin-like protein (Fragment) [*Lupinus albus*]	Q53HY0	57	26	62.0/6.08	1471	Central and C-terminal (154-521)
322	Conglutin β [*Lupinus albus*]	Q6EBC1	28	25	62.1/6.43	848	Central (64-268)
322	Conglutin γ [*Lupinus albus*]	Q9FSH9	6	30	49.9/8.39	157	Central (69-187)
323	Vicilin-like protein (Fragment) [*Lupinus albus*]	Q53HY0	6	21	62.0/6.08	118	
324	BLAD (Fragment) [*Lupinus albus*]	Q0R0N3	21	26	20.4/9.66	138	Central (98-139)
324	Conglutin γ [*Lupinus albus*]	Q9FSH9	11	26	49.9/8.39	72	
326	Vicilin-like protein (Fragment) [*Lupinus albus*]	Q53HY0	22	29	61.9/6.08	856	Central (101-451)
326	Conglutin γ [*Lupinus albus*]	Q9FSH9	6	29	49.9/8.39	86	Central (51-151)

331	Putative glutathione S-transferase [*Morus notabilis*]	W9RU43	12	24	25.2/5.50	322	N-terminal and Central (26-52)
332	Vicilin-like protein (Fragment) [Lupinus albus]	Q53HY0	28	25	61.9/6.08	765	Central (64-495)
334	Conglutin β precursor [*Lupinus albus*]	Q6EBC1	27	20	62.1/6.43	1395	Central and C-terminal (90-268/346-437)
334	Conglutin γ [*Lupinus albus*]	Q9FSH9	6	20	49.9/8.39	153	Central (69-187)
336	Conglutin β [*Lupinus albus*]	Q6EBC1	26	20	62.1/6.43	924	Central (90-268)
337	Conglutin β [*Lupinus albus*]	Q6EBC1	12	20	62.1/6.43	221	Central (154-404)
338	Legumin-like protein [*Lupinus albus*]	Q53I55	11	20	28.0/9.34	363	
339	NI			20			
341	Legumin-like protein [*Lupinus albus*]	Q53I54	18	20	59.0/5.53	706	Central (338-498)
341	Conglutin δ Seed storage protein [*Lupinus albus*]	Q333K7	20	20	17.6/5.47	273	C-terminal (101-131)
344	Conglutin β [*Lupinus albus*]	Q6EBC1	31	19	62.1/6.43	1383	Central (80-289)
344	Conglutin γ [*Lupinus albus*]	Q9FSH9	9	19	49.9/8.39	284	Central (68-279)
345	Legumin-like protein [*Lupinus albus*]	Q53I55	11	19	28.0/9.34	391	
346	Legumin-like protein [*Lupinus albus*]	Q53I54	18	19	50.0/5.53	689	Central (338-498)
350	Conglutin δ Seed storage protein [*Lupinus albus*]	Q333K7	41	17	17.6/5.47	450	Central and C-terminali (75-139)
350	Vicilin-like protein (Fragment) [*Lupinus albus*]	Q53HY0	11	17	62.0/6.08	349	Central (427-496)
351	Vicilin-like protein (Fragment) [Lupinus albus]	Q53HY0	26	18	62.0/6.08	893	Central (194-463)
353	Vicilin-like protein (Fragment) [*Lupinus albus*]	Q53HY0	21	17	62.0/6.08	569	Central and C-terminal (275-521)
354/352	Conglutin β [*Lupinus albus*]	Q6EBC1	15	18	62.1/6.43	421	Central (274-496)
355	Conglutin δ Seed storage protein [*Lupinus albus*]	Q333K7	35	18	17.6/5.47	363	Central and C-terminal (75-131)
356	Conglutin β [*Lupinus albus*]	Q6EBC1	16	16	62.1/6.43	569	Central (324-495)

357	HSP18.1A [*Citrullus lanatus*]	H6TB42	30	16	18.1/6.35	387	Central (67-144)
357	Conglutin γ [*Lupinus albus*]	Q9FSH9	14	16	49.9/8.39	322	Central (324-427)
358	Conglutin γ [*Lupinus albus*]	Q9FSH9	15	15.7	49.9/8.39	322	
359	Conglutin α 2 [*Lupinus angustifolius*]	F5B8V7	2	17	74.6/5.12	91	C-terminal (598-611)
360	Conglutin γ [*Lupinus albus*]	Q9FSH9	6	18	49.9/8.39	146	Central (69-187)
361	Conglutin β [*Lupinus albus*]	Q6EBC1	20	18	62.1/6.43	686	Central (316-495)
362	17.5 kd heat shock family protein [*Populus trichocarpa*]	B9IQG7	33	17	17.5/5.98	599	Central (67-141)
363/370	Vicilin-like protein (Fragment) [*Lupinus albus*]	Q53HY0	49	16	62.0/6.08	1213	N-terminal and Central (45-495)
363/370	Conglutin γ [*Lupinus albus*]	Q9FSH9	30	16	49.9/8.39	702	Central (68-426)
364	Vicilin-like protein (Fragment) [*Lupinus albus*]	Q53HY0	19	17	62.0/6.08	674	
364	Conglutin δ Seed storage protein [*Lupinus albus*]	Q333K7	35	17	17.6/5.47	299	Central and C-terminal (75-131)
368	Seed storage protein [*Lupinus angustifolius*]	F5B8V8	14	16	57.25/5.4	984	C-terminal (320-469)
368	Conglutin γ [*Lupinus albus*]	Q9FSH9	20	19	49.9/8.39	436	
369	Conglutin γ [*Lupinus albus*]	Q9FSH9	19	16.5	49.9/8.39	720	C-terminal (324-452)
372	Conglutin γ [*Lupinus albus*]	Q9FSH9	26	17	49.9/8.39	1050	Central and C-terminal (324-452)
373/384	Conglutin δ Seed storage protein [*Lupinus albus*]	Q333K7	41	13	17.6/5.47	514	Central and C-terminal (75-140)
373/384	Vicilin-like protein (Fragment) [*Lupinus albus*]	Q53HY0	11	13	62.0/6.08	286	Central (427-496)
374	BLAD (Fragment) [*Lupinus albus*]	Q0R0N3	9	15	20.4/9.66	107	Central (98-114)
374	Conglutin γ [*Lupinus albus*]	Q9FSH9	2	17	49.9/8.39	70	C-terminal (442-452)
375	Conglutin γ [*Lupinus albus*]	Q9FSH9	26	16	49.9/8.39	954	
376	Conglutin γ [*Lupinus albus*]	Q9FSH9	65	16	49.9/8.39	829	Central and C-terminal (59-452)

376	Vicilin-like protein (Fragment) [*Lupinus albus*]	Q53HY0	28	16	62.0/6.08	768	
377	Conglutin γ [*Lupinus albus*]	Q9FSH9	36	18	49.9/8.39	1140	Central and C-terminal (162-452)
378	Conglutin γ [Lupinus albus]	Q9FEX1	31	16	50.2/6.61	863	Central and C-terminal (303-448)
381	Vicilin-like protein (Fragment) [*Lupinus albus*]	Q53HY0	37	14	62.0/6.08	1045	Central (206-473)
381	Conglutin δ Seed storage protein [*Lupinus albus*]	Q333K7	35	14	17.6/5.47	417	Central and C-terminal (75-131)
383	Vicilin like protein [*Lupinus albus*]	Q53HY0	12	14.5	62.0/6.08	731	Central (206-473)
385	Conglutin δ [*Lupinus albus*]	Q333K7	35	15	17.6/5.47	394	Central and C-terminal (75-131)
386	Vicilin-like protein (Fragment) [*Lupinus albus*]	Q53HY0	27	13	62.0/6.08	840	Central (154-463)
386	Conglutin δ Seed storage protein [*Lupinus albus*]	Q333K7	35	13	17.6/5.47	438	Central and C-terminal (75-131)
387	Conglutin δ Seed storage protein [*Lupinus albus*]	Q333K7	35	14	17.6/5.47	421	Central and C-terminal (75-131)
388	Conglutin δ [*Lupinus albus*]	Q333K7	20	13	17.6/5.47	289	C-terminal (101-131)
388	Conglutin γ [*Lupinus albus*]	Q9FSH9	17	13	49.9/8.39	261	
389	Vicilin-like protein (Fragment) [*Lupinus albus*]	Q53HY0	26	18	62.0/6.08	893	Central (194-463)
389	Conglutin δ Seed storage protein [*Lupinus albus*]	Q333K7	35	18	17.6/5.47	352	Central and C-terminal (75-131)
389	Conglutin γ [*Lupinus albus*]	Q9FSH9	10	18	49.9/8.39	162	Central (379-426)

Table S2. List of the identified protein spots with differences in relative abundances of Modica Ecotype *vs* Luxor cultivar comparison. Spots numbers are referred to **Figure S8.**

Spot	Protein [Organism]	Accession Number	Coverage %	Exp Mr	Theor Mr/pI	Matched Peptides	Unique Peptides	MASCOT score	ANOVA	Fold	Sequence region
31*	vicilin-like protein [Lupinus albus]	Q53HY0	26	30	62/6.08	11	2	732	9.110E-04	0.202	
	beta-conglutin precursor [Lupinus albus]	Q6EBC1	23	30	62.1/6.43	11	2	706			N-terminal and Central
	conglutin gamma [Lupinus albus	Q9FSH9	11	30	49.9/8.39	5	3	404			N-terminal and Central
	conglutin gamma [Lupinus albus]	Q9FEX1	6	30	50.2/6.61	3	1	346			
	conglutin gamma 2 [Lupinus angustifolius]	F5B8W7	7	30	47.9/6.42	3	1	282			N-terminal and Central
	conglutin beta 6 [Lupinus angustifolius]	F5B8W4	6	30	70.1/6.18	3	1	100			Central
41*	vicilin-like protein [Lupinus albus]	Q53HY0	27	35	62/6.08	12	3	1279	1.200E-03	0.315	Central
43*	vicilin-like protein [Lupinus albus]	Q53HY0	41	40	62/6.08	24	8	2293	2.510E-04	0.243	Central
	beta-conglutin precursor [Lupinus albus]	Q6EBC1	34	40	62.1/6.43	19	3	1926			Central
49*	conglutin alpha 2 [Lupinus angustifolius]	F5B8V7	6	42	74.6/5.12	2	1	268	1.500E-03	2.253	C-terminal
	seed storage protein [Lupinus angustifolius]	F5B8V8	7	42	57.2/5.45	2	1	189			C-terminal
	conglutin delta seed storage protein precursor [Lupinus albus]	Q333K7	20	42	17.6/5.47	2	2	150			C-terminal
	putative TAG factor protein [Lupinus angustifolius]	AAN15136	9	42	32.5/6.33	2	2	86			

Table S3. List of identified proteins resulting exclusive of Modica Ecotype in the comparison with Luxor cv. Spots numbers are referred to **Figure S8**.

Spot	Protein [Organism]	Accession Number	Coverage %	Exp.Mr	Theor. Mr/pI	Matched Peptides	Unique Peptides	MASCOT score	ANOVA	NOTE

72*	NI			10						
75*	NI			15						
84*	1-Cys peroxiredoxin-like [Glycine max]	XP_003531110	13	25	24.5 /6.4	3	3	346	2.360E-04	Central
	beta-conglutin precursor [Lupinus albus]	Q6EBC1	9	25	62.1 /6.4	4	4	268		Central
	1-Cys peroxiredoxin [Medicago truncatula]	REHY_MEDTR	13	25	24.6 /6.0	3	3	185		N-terminal and Central
112*	conglutin delta seed storage protein precursor [Lupinus albus]	Q333K7	20	15	17.6 /5.4	2	2	290	9.260E-03	C-terminal
	conglutin alpha 2 [Lupinus angustifolius]	F5B8V7	6	15	74.6 /5.1	2	2	223		C-terminal

Table S4. List of identified proteins resulting exclusive of Luxor cv in the comparison with Modica Ecotype. Spots numbers are referred to **Figure S8**.

Spot	Protein [Organism]	Accession Number	Coverage %	Exp.Mr	Theor.Mr/pI	Matched Peptides	Unique Peptides	MASCOT score	ANOVA	NOTE
125*	vicilin-like protein [Lupinus albus]	Q53HY0	12	14.5	62.0/6.08	8	6	731	3.150E-05	Central
	beta-conglutin precursor [Lupinus albus]	Q6EBC1	9	14.5	62.1/6.43	4	2	379		C-terminal
129*	conglutin gamma [Lupinus albus]	Q9FSH9	9	27	49.9/8.39	3	2	291	4.040E-04	
	beta-conglutin precursor [Lupinus albus]	Q6EBC1	11	27	62.1/6.43	5	5	195		
	1-Cys peroxiredoxin-like [Glycine max]	XP_003531110	13	27	24.5/6.44	3	3	160		Central
	1-Cys peroxiredoxin [Medicago truncatula]	REHY_MEDTR	13	27	24.6/6.08	2	2	134		N-terminal and Central

Table S5. List of identified proteins resulting exclusive of Taper cv in the Dukat vs Taper cultivar comparison. Spots numbers are referred to **Figure S9**.

**Spot**	**Protein [organism]**	**Accession Number**	**Cover age %**	**Exp. Mr.**	**Theor. Mr/pI**	**Matched peptides**	**Unique peptides**	**MASCOT score**	**ANOVA**	**Sequence region**
73*	Conglutin alpha 3 [Lupinus angustifolius]	F5B8V8	13	17	67.7/5.31	43(14)	16	582	4.5E-3	Central
74*	Seed storage protein [Lupinus angustifolius]	F5B8V8	19	17	57.2/5.45	403(230)	49	648	9.8E-3	Central and C-terminal
	Conglutin gamma [Lupinus angustifolius]	Q42369	3		49.6/7.66	1(1)	1	57		Central
76*	NI			22					6.4E-4	
78*	Conglutin alpha 1 [Lupinus angustifolius]	F5B8V6	15	20	58.2/5.26	222(95)	25	566	6.6E-3	Central and C-terminal
79*	1-Cys peroxiredoxin [Glycine soja]	A0A0B2RB28	8	25	24.5/6.90	13(2)	13	67	3.3E-5	Central
	Uncharacterized protein [Lotus japonicas]	I3SM62	9		24.8/6.12	7(2)	7	64		N-terminal
80*	NI	-	-	26	-	-	-	-	4.5E-3	

Table S6. List of identified proteins resulting exclusive of Dukat cv in the Dukat vs Taper cultivar comparison. Spots numbers are referred to **Figure S9**.

**Spot**	**Protein [organism]**	**Accession Number**	**Cover age %**	**Exp. Mr.**	**Theor. Mr/pI**	**Matched peptides**	**Unique peptides**	**MASCOT score**	**ANOVA**	**Sequence region**
88*	NI	-	-	20	-	-		-	3.3E-3	
99*	Annexin-like protein RJ4 [Glycine soja]	A0A0B2QZN2	14	30	35.8/7.12	12(4)	3	223	6.2E-5	Central
	Putative TAG factor protein [Lupinus angustifolius]	Q8GSE7	13		32.5/6.33	8(6)	1	201		Central

Table S7. List of identified proteins resulting exclusive of Taper cv in the Mister vs Taper cultivar comparison. Spots numbers are referred to **Figure S10**

Spot	Protein [organism]	Accession Number	Coverage %	Exp. Mr.	Theor. Mr/pI	Matched peptides	Unique peptides	MASCOT score	ANOVA	Sequence region
66*	Annexin [Medicago truncatula]	G7KB73	14	30	36.3/7.14	26(7)	7	301	6.8E-4	
	Annexin D1 [Glycine soja]	A0A0B2SDH4	12		36.1/6.48	18(7)	1	280		
	Annexin [Arabis alpine]	A0A087G1V1	11		36.3/5.29	10(3)	-	167		Central (87-267)
	Annexin [Brassica napus]	A0A078CEH0	11		36.2/5.27	5(2)	1	155		
	Annexin [Theobroma cacao]	A0A061DJJ7	12		36.0/6.22	5(1)	3	128		
	Conglutin beta 5 [Lupinus angustifolius]	F5B8W3	5		75.2/5.75	2(0)	1	95		Central (307-381)
	Annexin [Phaseolus vulgaris]	V7B5V0	3		35.9/8.23	5(3)	2	69		Central (64-75)

Table S8. List of identified proteins resulting exclusive of Mister cv in the Mister vs Taper cultivar comparison. Spots numbers are referred to **Figure S10**

Spot	Protein [organism]	Accession Number	Coverage %	Exp. Mr.	Theor. Mr/pI	Matched peptides	Unique peptides	MASCOT score	ANOVA	Sequence region
81*	Conglutin alpha 1 [Lupinus angustifolius]	F5B8V6	7	12	58.1/5.26	8(3)	5	135	4.6E-4	C-terminal (447-483)
	BnaA10g19320D protein [Brassica napus]	A0A078C3J4	3		50.3/8.61	14(2)	2	67		Centrali (266-281)
82*	Uncharacterized protein [Jatropha curcas]	A0A067K1S4	2	12	55.0/9.26	9(1)	3	70	1.8E-4	C-terminal (445-458)
83*	Conglutin delta 1 [Lupinus angustifolius]	F5B8W8	20	12	18.2/5.47	9(3)	6	171	1.3E-4	C-terminal (105-135)
	Kinase, putative [Ricinus communis]	B9RES6	1		90.5/5.29	34(0)	3	170		C-terminal (707-721)
	Conglutin alpha 1 [Lupinus angustifolius]	F5B8V6	7		58.1/5.26	13(10)	3	136		C-terminal (467-493)
	Uncharacterized protein [Brassica rapa subsp. Pekinensis]	M4EZI2	6		35.1/7.68	115(1)	3	61		Central (201-218)
85*	Conglutin delta 1 [Lupinus angustifolius]	F5B8W8	20	15	18.2/5.47	11(9)	8	169	7.2E-4	Central (105-135)
	Conglutin alpha 1 [Lupinus angustifolius]	B0YJF7	7		58.1/5.26	2(1)	2	122		Central (447- 483)
	Kinesin-like protein [Arabidopsis thaliana]	V7ALP7	3		66.4/9.59	3(0)	2	84		
92*	Conglutin alpha 1 [Lupinus angustifolius]	F5B8V6	15	24	58.1/5.26	295(131)	31	531	5.9E-3	
	Legumin-like protein [Lupinus albus]	Q53I54	12		58.8/5.33	74(17)	4	244		Central and C-terminal (338-519)
96*	Putative TAG factor protein [Lupinus angustifolius]	Q8GSE7	13	30	32.5/6.33	11(9)	2	216	8.5E-3	

Conglutin beta (Fragment) [Lupinus angustifolius]	B0YJF7	7	61.5/5.34	5(1)	-	148	Central (131-149)
Annexin [Phaseolus vulgaris]	V7ALP7	7	35.9/8.57	4(4)	-	146	Central (64-156)
Annexin-like protein RJ4 [Glycine soja]	A0A0B2QZN2	6	35.8/7.12	5(3)	-	127	

Table S9. List of the identified protein spots in Sonet cultivar of *Lupinus angustifolius.* Spots are referred to **Figure 3B**.

**Spot**	**Protein [organism]**	**Accession Number**	**Cover age %**	**Exp Mr.**	**Theor. Mr/pI**	**MASCOT score**	**Sequence region (amino acid posistion)**
161	Conglutin β 5 [*Lupinus angustifolius*]	F5B8W3	26	40	75.2/5.75	1073	Central (103-375)
166	Conglutin β [*Lupinus angustifolius*]	B0YJF8	24	40	54.3/6.27	576	Central (214-339)
177	Conglutin β [*Lupinus angustifolius*]	B0YJF8	32	40	54.3/6.27	1119	Central (111-339)
192	Conglutin β [*Lupinus angustifolius*]	B8Q5G0	20	38	71.8/5.82	683	Central (214-591)
194	Conglutin β 1 [*Lupinus angustifolius*]	F5B8V9	26	30	71.9/5.82	1016	Central (111-538)
195	Conglutin β 2 [*Lupinus angustifolius*]	F5B8W0	36	39	70.6/5.64	1227	Central and C-terminal (175-576)
205	Conglutin β 2 [*Lupinus angustifolius*]	F5B8W0	28	30	70.6/5.64	1164	Central (82-573)
214	Conglutin β 2 [*Lupinus angustifolius*]	F5B8W0	31	29	70.6/5.64	1090	Central and C-terminal (113-576)
218	Conglutin β 4 [*Lupinus angustifolius*]	F5B8W2	36	29	69.7/5.96	1274	Central and C-terminal (172-570)
224	Conglutin β 4 [*Lupinus angustifolius*]	F5B8W2	40	29	69.4/5.96	1226	Central and C-terminal (202-570)
226	Conglutin β 4 [*Lupinus angustifolius*]	F5B8W2	47	29	69.4/5.96	1470	Central and C-terminal (170-570)
228	Conglutin β 2 [*Lupinus angustifolius*]	F5B8W0	4	29	70.6/5.64	155	Central (511-525)
231	Conglutin β 3 [*Lupinus angustifolius*]	F5B8W1	33	29	68.1/5.71	1064	Central and C-terminal (160-560)
255	Conglutin β 2 [*Lupinus angustifolius*]	F5B8W0	12	25	70.6/5.64	425	Central (173-535)
257	Conglutin β 4 [*Lupinus angustifolius*]	F5B8W2	20	25	69.4/5.96	397	Central and C-terminal (213-570)
262	Conglutin β 4 [*Lupinus angustifolius*]	F5B8W2	41	25	69.4/5.96	1256	Central and C-terminal (170-570)
265	Conglutin β 4 [*Lupinus angustifolius*]	F5B8W2	37	25	69.4/5.96	1169	Central and C-terminal (170-570)
271	Conglutin β 5 [*Lupinus angustifolius*]	F5B8W3	20	25	75.2/5.75	828	Central (213-376)
271	Conglutin α 1 [*Lupinus angustifolius*]	F5B8V6	17	25	58.2/5.26	501	
273	Conglutin β 4 [*Lupinus angustifolius*]	F5B8W2	18	25	69.4/5.96	705	Central (170-327)
274	Conglutin β 2 [*Lupinus angustifolius*]	F5B8W0	24	25	70.7/5.64	791	

274	Conglutin α 1 [*Lupinus angustifolius*]	F5B8V6	11	25	58.2/5.26	273	Central and C-terminal (354-483)
275	Conglutin α 1 [*Lupinus angustifolius*]	F5B8V6	22	23	58.2/5.26	821	Central and C-terminal (332-503)
275	Conglutin β 2 [*Lupinus angustifolius*]	F5B8W0	22	23	70.7/5.64	770	Central (172-330)
279/281/297/295	Conglutin α 1 [*Lupinus angustifolius*]	F5B8V6	22	20	58.2/5.26	10246	Central and C-terminal (332-503)
279/281/297/295	Conglutin β 3 [*Lupinus angustifolius*]	F5B8W1	25	20	68.1/5.71	7767	Central (160-507)
280	Conglutin β 4 [*Lupinus angustifolius*]	F5B8W2	47	20	69.4/5.96	1325	Central and C-terminal (170-570)
287	Conglutin α 1 [*Lupinus angustifolius*]	F5B8V6	20	20	58.2/5.26	11477	Central and C-terminal (332-503)
287	Conglutin β 2 [*Lupinus angustifolius*]	F5B8W0	22	20	70.7/5.64	722	Central (172-330)
307	Conglutin α 2 [*Lupinus angustifolius*]	F5B8V7	15	18	74.6/5.12	3783	Central and C-terminal (468-619)
308	Conglutin α 2 [*Lupinus angustifolius*]	F5B8V7	23	17	74.6/5.12	11127	Central and C-terminal (468-629)
308	Conglutin β (Fragment) [*Lupinus angustifolius*]	B0YJF7	19	17	61.5/5.34	387	Central (128-243)
314/324	Conglutin α 3 [*Lupinus angustifolius*]	F5B8V8	26	17	57.2/5.45	577	Central and C-terminal (143-459)
316	Conglutin α 3 [*Lupinus angustifolius*]	F5B8V8	23	18	57.2/5.45	8173	Central and C-terminal (320-462)
318/319/328	Conglutin β 4 [*Lupinus angustifolius*]	F5B8W2	26	17	69.4/5.96	625	Central and C-terminal (202-570)
323	Conglutin β (Fragment) [*Lupinus angustifolius*]	B0YJF7	18	17	61.5/5.34	649	N-terminal and Central (29-209)
325	Conglutin β 1 [*Lupinus angustifolius*]	F5B8V9	23	17	71.9/5.82	807	
326	Conglutin β 1 [*Lupinus angustifolius*]	F5B8V9	24	17	71.9/5.82	886	
331	Chaperone [*Agave tequilana*]	A4ZF48	47	17	18.4/6.78	671	Central and C-terminal (56-161)
332	Conglutin β (Fragment) [*Lupinus angustifolius*]	B0YJF7	24	17	61.5/5.34	750	
334	Conglutin β 2 [*Lupinus angustifolius*]	F5B8W0	30	16	70.6/5.64	730	Central and C-terminal (113-576)
336/339/345	Conglutin γ [*Lupinus angustifolius*]	Q42369	22	16	49.6/7.66	794	Central and C-terminal (343-450)
337	Conglutin γ [*Lupinus angustifolius*]	Q42369	22	16	49.6/7.66	23614	Central and C-terminal (342-450)
338/348	Conglutin β 2 [*Lupinus angustifolius*]	F5B8W0	37	16	70.7/5.64	1083	Central and C-terminal (111-576)

346	Conglutin β 2 [*Lupinus angustifolius*]	F5B8W0	8	15	70.6/5.64	257	Central (112-319)
346	Conglutin γ [*Lupinus angustifolius*]	Q42369	15	15	49.6/7.66	229	Central and C-terminal (355-423)
356	Conglutin γ [*Lupinus angustifolius*]	Q42369	44	10	49.6/7.66	722	Central and C-terminal (67-450)
